# Involvement of the Retinoid X Receptor Ligand in the Anti-Inflammatory Effect Induced by Peroxisome Proliferator-Activated Receptor **γ** Agonist In Vivo

**DOI:** 10.1155/2011/840194

**Published:** 2011-12-11

**Authors:** Atsuki Yamamoto, Hiroki Kakuta, Hiroyuki Miyachi, Yukio Sugimoto

**Affiliations:** Okayama University Graduate School of Medicine, Dentistry and Pharmaceutical Sciences, Tsushima-naka 1-1-1, Kita-ku, Okayama 700-8530, Japan

## Abstract

Peroxisome proliferator-activated receptor **γ** (PPAR**γ**) forms a heterodimeric DNA-binding complex with retinoid X receptors (RXRs). It has been reported that the effect of the PPAR agonist is reduced in hepatocyte RXR-deficient mice. Therefore, it is suggested that the endogenous RXR ligand is involved in the PPAR**γ** agonist-induced anti-inflammatory effect. However, the participation of the RXR ligand in the PPAR**γ**-induced anti-inflammatory effect is unknown. Here, we investigated the influence of RXR antagonist on the anti-inflammatory effect of PPAR**γ** agonist pioglitazone in carrageenan test. In addition, we also examined the influence of PPAR antagonist on the anti-inflammatory effect induced by RXR agonist NEt-3IP. The RXR antagonist suppressed the antiedema effect of PPAR**γ** agonist. In addition, the anti-inflammatory effect of RXR agonist was suppressed by PPAR**γ** antagonist. PPAR**γ** agonist-induced anti-inflammatory effects were reversed by the RXR antagonist. Thus, we showed that the endogenous RXR ligand might contribute to the PPAR**γ** agonist-induced anti-inflammatory effect.

## 1. Introduction

Peroxisome proliferator-activated receptor (PPAR) is a family comprising 3 different isoforms: PPAR*α*, PPAR*γ*, and PPAR*δ*. PPAR forms a heterodimeric DNA-binding complex with the retinoid X receptor (RXR) and serves as a transcriptional regulator of genes involved in lipid metabolism [1, 2]. In addition, it has been reported that PPAR*γ* is expressed in monocytes and macrophages; therefore, researchers have shown much interest in the involvement of PPAR*γ* in inflammatory processes [3–6]. Studies have shown that the PPAR*γ* agonist is effective in inflammatory models such as intestinal inflammation [7], rheumatoid arthritis [8], inflammatory lung disease [9], and allergic rhinitis [10]. These studies suggested that PPAR*γ* agonist might be a new drug for the treatment of inflammatory disease.

RXR is a member of the nuclear hormone receptor superfamily and is activated by the endogenous agonist 9-cis retinoic acid [11]. RXR functions as a dimer not only with PPAR but also with other nuclear receptor partners such as retinoid acid receptor (RAR), vitamin D receptor (VDR), and liver X receptor (LXR) [2, 12]. Therefore, RXR is closely linked to the function of such partners, and RXR agonists synergistically control the function of RXR heterodimeric partners [13]. Manzano et al. [14] have reported that in human mesangial cells, 9-cis retinoic acid suppressed the expression of vascular cell adhesion molecule (VCAM)-1 and intercellular adhesion molecule (ICAM)-1 that was induced by lipopolysaccharide (LPS), a bacterial endotoxin. In addition, it has been reported that in microglial cells, 9-cis retinoic acid reduced tumour necrosis factor-*α* (TNF-*α*)-induced nitric oxide (NO) expression [15]. Therefore, it is suggested that the RXR agonist exerts an anti-inflammatory effect. However, 9-cis retinoic acid activated not only RXR but also RAR. Motomura et al. [16] have reported that the suppressive effect of 9-cis retinoic acid was not reversed by RAR-specific antagonist. Moreover, the RAR-specific agonist Ro 40-6055 did not show the inhibitory effect shown by 9-cis retinoic acid on the increase of NO and TNF-*α* levels in Kupffer cells [16]. Therefore, it is suggested that the inhibitory effect of 9-cis retinoic acid does not depend on the RAR/RXR signalling pathway but on another RXR heterodimer signalling pathway. Benson et al. [17] have reported that the antiproliferative activity induced by the endogenous PPAR agonist 15 deoxy-Δ^12,14^-PGJ_2_ was enhanced by the endogenous RXR agonist 9-cis retinoic acid. In addition, coactivation of the PPAR*γ* agonist troglitazone and the RXR agonist LG100268 resulted in additive effects on glucose and lipid metabolism in skeletal muscles [18]. Moreover, Diab et al. [15] have reported that in microglial cells, 15 deoxy-Δ^12,14^-PGJ_2_ and 9-cis retinoic acid individually weakly inhibited NO production but together strongly and synergistically inhibited NO production. Furthermore, it has been reported that PPAR agonist did not inhibit carrageenan-induced paw edema in hepatocyte-specific RXR-deficient mice, whereas PPAR agonist reduced carrageenan-induced paw edema in wild-type mice [19]. Therefore, we hypothesized that the endogenous RXR ligand is involved in the PPAR agonist-induced anti-inflammatory effect. However, it is not known whether PPAR was activated by RXR in a ligand-dependent manner.

Carrageenan-induced paw edema has been increasingly used to test new anti-inflammatory drugs as well as to study the mechanisms involved in inflammation. Therefore, carrageenan-induced local inflammation is a useful model to assess the contribution of mediators involved in vascular changes associated with acute inflammation [20–23]. In the present study, we examined the effect of the RXR antagonist on the anti-inflammatory effect of the PPAR*γ* agonist pioglitazone in order to investigate the participation of RXR in PPAR*γ* activation. Moreover, we examined the effects of PPAR*α*, PPAR*γ*, and PPAR*δ* antagonists on the anti-inflammatory effect induced by the RXR agonist NEt-3IP with the aim of determining the PPAR subtype that is involved in the RXR agonist-induced anti-inflammatory effect.

## 2. Materials and Methods

### 2.1. Animals

Five-week-old male ICR mice (body weight, 23–28 g) were purchased from Japan SLC, Shizuoka, Japan. The animals were kept in an air-conditioned room at a controlled temperature (24°C ± 2°C) and humidity (55%  ± 15%). They were housed in plastic cages lined with sawdust and kept under a light-dark cycle (lights on from 0700–1900). Food and water were freely available, except during test periods. All procedures involving animals were conducted in accordance with the Guidelines for Animal Experiments at Okayama University Advanced Science Research Center, and all procedures were licensed by the Animal Research Control Committee of Okayama University.

### 2.2. Reagents

*λ*-Carrageenan (Wako, Osaka, Japan) was dissolved in physiological saline. 6-[*N*-ethyl-*N*-(3-isopropoxy-4-isopropylphenyl)-amino] nicotinic acid (NEt-3IP), 6-[*N*-*4*-(trifluoromethyl) benzenesulfonyl-*N*-(5,5,8,8-tetramethyl-5,6,7,8-tetrahydro-2-naphthyl) amino] nicotinic acid (NS-4TF), and 3′-((2-fluoro-4-(trifluoromethyl) benzamido) methyl)-4′-propoxybiphenyl-4-carboxylic acid (JKPL-85) were synthesized at Okayama University [24, 25]. NEt-3IP and pioglitazone (Actos; Takeda Pharmaceutics, Osaka, Japan) were suspended in 0.5% carboxymethylcellulose solution. Bisphenol A diglycidyl ether (BADGE; Sigma, St. Louis, Mo, USA), *N*-((2*S*)-2-(((1*Z*)-1-methyl-3-oxo-3-(4-(trifluoromethyl) phenyl) prop-1-enyl) amino)-3-(4-(2-(5-methyl-2-phenyl-1,3-oxazol-4-yl) ethoxy) phenyl) propyl) propanamide (GW6471; Sigma), 2-chloro-5-nitro-*N*-phenylbenzamide (GW9662; Sigma), and JKPL-85 were dissolved in physiological saline containing 10% dimethylsulphoxide. Actinomycin D (Sigma) was dissolved in physiological saline containing 10% ethanol.

### 2.3. Drug Administration

Both pioglitazone (1, 3, and 10 mg/kg) and NEt-3IP (1, 3, and 10 mg/kg) were orally administered 3 h before carrageenan injection. NS-4TF (10 and 30 *μ*g/paw), GW6471 (10 and 30 *μ*g/paw), GW9662 (1, 3, and 10 *μ*g/paw), JKPL-85 (10 and 30 *μ*g/paw), and actinomycin D (3 and 10 *μ*g/paw) were injected into the subplantar region of the hind paw 15 min before carrageenan injection. BADGE (10 and 30 mg/kg) was intraperitoneally injected into mice 30 min before the carrageenan injection.

### 2.4. Mouse Paw Edema

After carrageenan injection, the hind paw volume was measured at intervals of 1 h for up to 3 h. The paw volume was determined using a plethysmometer (TK-101; UNICOM, Chiba, Japan). The basal volume of the hind paw was determined before the administration of any drug. After determination of the basal volume, the animals were divided into experimental groups in such a way that the mean volumes of the different groups were similar. A 1% solution of *λ*-carrageenan dissolved in saline (0.05 mL/animal) was injected subcutaneously into the right hind paw of each mouse. Paw edema was determined as the difference in the paw volume before and after carrageenan injection and was expressed as Δ paw volume.

### 2.5. Statistical Analysis

All data are presented as the mean ± standard error of the mean (S.E.M.). Statistical analysis was performed using one-way analysis of variance (ANOVA) with the Dunnett's test or Student's unpaired *t* test. When the probability (*P*) value was less than 0.05, the difference was considered to be significant.

## 3. Results

### 3.1. Anti-Inflammatory Effect of PPAR*γ* Agonist

**Figure 1:**
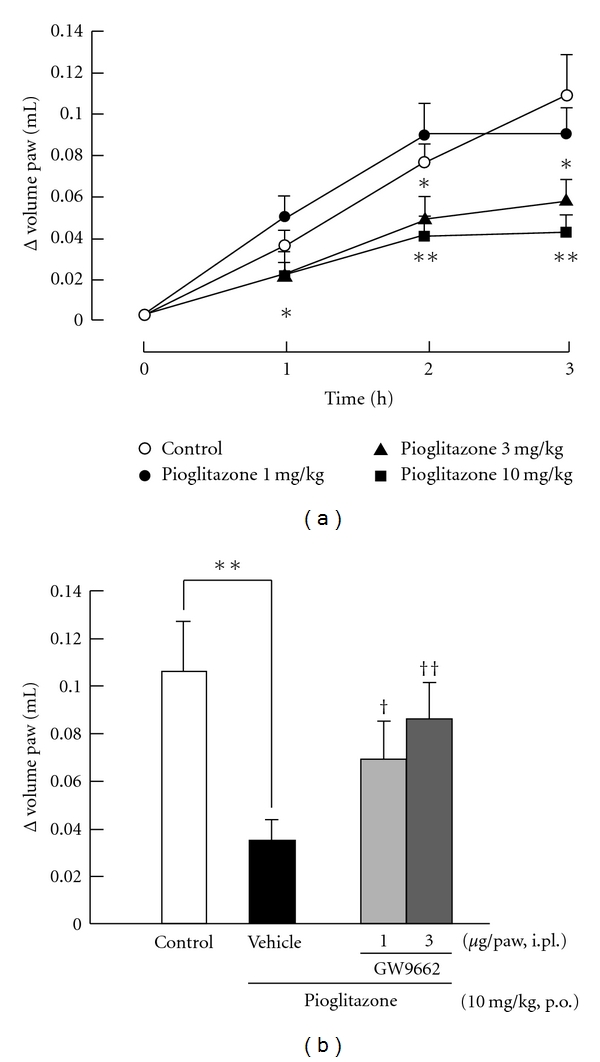
*Involvement of PPAR*γ* on carrageenan-induced paw edema in mice.* (a) Dose-dependence and time course of PPAR*γ* agonist, pioglitazone on carrageenan-induced paw edema in mice. Pioglitazone was orally administrated 3 h before carrageenan injection at doses of 1, 3, and 10 mg/kg. The control group received 0.5% carboxymethylcellulose. (b) Effect of PPAR*γ* antagonist, GW9662 on pioglitazone induced anti-inflammatory effect. Pioglitazone was orally administrated 3 h before carrageenan injection at a dose of 10 mg/kg. GW9662 was injected 15 min before carrageenan injection at doses of 1 and 3 *μ*g/paw. The vehicle group received physiological saline including 10% dimethylsulphoxide. Each column and vertical bar represents the means ± S.E.M. (*n* = 7). *,**: Significantly different from the control group at *P* < 0.05 and *P* < 0.01, respectively, (Dunnett's test). ^†^,^††^: Significantly different from the vehicle group at *P* < 0.05 and *P* < 0.01, respectively, (Dunnett's test).

The PPAR*γ* agonist pioglitazone showed anti-inflammatory effect in a dose-dependent manner (Figure 1(a)). Oral administration of pioglitazone at doses of 3 and 10 mg/kg significantly inhibited carrageenan-induced paw edema as compared with that of the control group. Pioglitazone (3 mg/kg) significantly suppressed paw edema at 2 and 3 h after carrageenan injection. Pioglitazone (10 mg/kg) significantly suppressed paw edema at 1, 2, and 3 h after carrageenan injection.  Figure 1(b) shows the effect of the PPAR*γ* antagonist GW9662 on the anti-inflammatory effect of pioglitazone. Intraplantar injection of GW9662 at doses of 1 and 3 *μ*g/paw significantly reduced the anti-inflammatory effect of pioglitazone.

### 3.2. Influence of RXR Antagonists on the Anti-Inflammatory Effect Induced by PPAR*γ* Agonist

**Figure 2:**
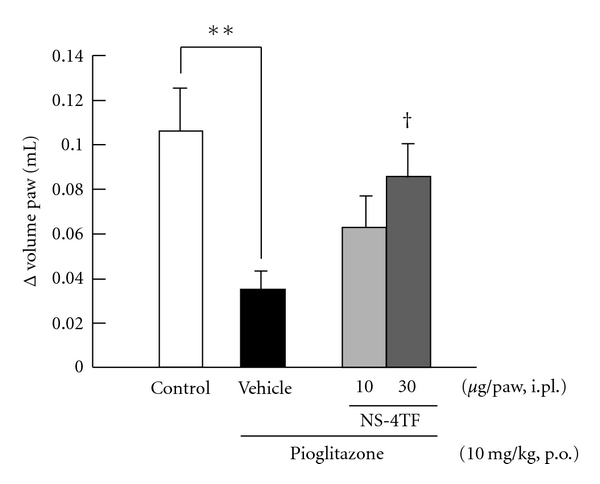
*Influence of RXR antagonists on PPAR*γ* agonist-induced anti-inflammatory effects*. Pioglitazone was orally administrated 3 h before carrageenan injection at a dose of 10 mg/kg. RXR antagonist, NS-4TF was injected into subplantar 15 min before carrageenan injection at doses of 10 and 30 *μ*g/paw. The vehicle group received physiological saline including 10% dimethylsulphoxide. Each column and vertical bar represents the means ± S.E.M. (*n* = 7). **: Significantly different from the control group at *P* < 0.01 (Dunnett's test). ^†^: Significantly different from the vehicle group at *P* < 0.05 (Dunnett's test).

Figure 2 shows the influence of NS-4TF on the anti-inflammatory effect of pioglitazone. Intraplantar injection of NS-4TF at a dose of 30 *μ*g/paw significantly reduced the anti-inflammatory effect induced by pioglitazone.

### 3.3. Anti-Inflammatory Effect of RXR Agonist

**Figure 3:**
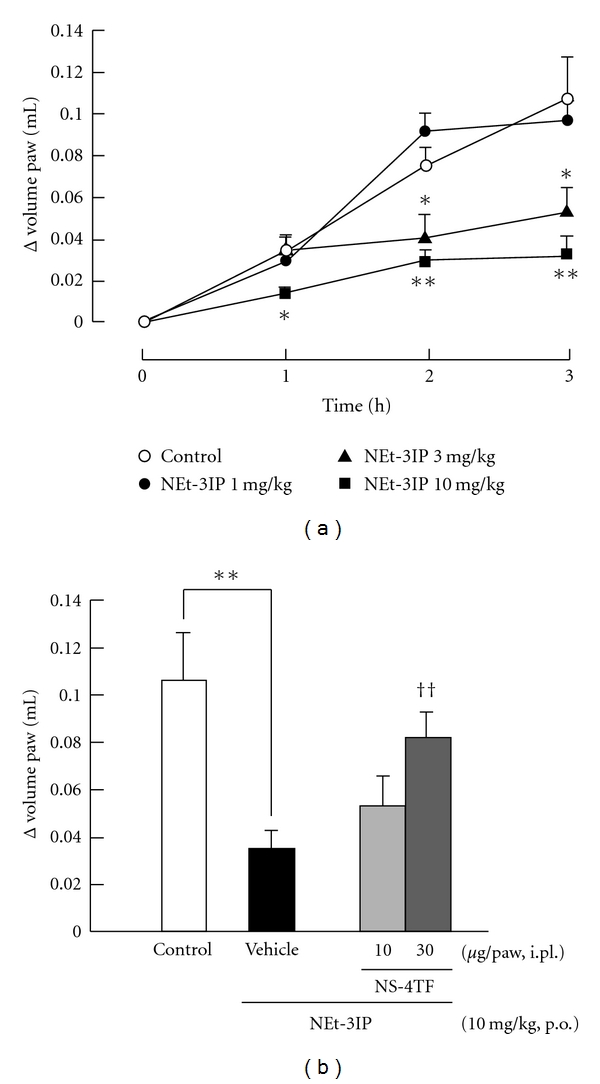
*Involvement of RXR on carrageenan-induced paw edema in mice*. (a) Dose dependence and time course of RXR agonist, NEt-3IP on carrageenan-induced paw edema in mice. NEt-3IP was orally administrated 3 h before carrageenan injection at doses of 1, 3, and 10 mg/kg. The control group was received 0.5% carboxymethylcellulose. (b) Effect of RXR antagonist, NS-4TF on NEt-3IP induced anti-inflammatory effect. NEt-3IP was orally administrated 3 h before carrageenan injection at a dose of 10 mg/kg. NS-4TF was injected 15 min before carrageenan injection at doses of 10 and 30 *μ*g/paw. The vehicle group received physiological saline including 10% dimethylsulphoxide. Each column and vertical bar represents the means ± S.E.M. (*n* = 7). *,**: Significantly different from the control group at *P* < 0.05 and *P* < 0.01, respectively, (Dunnett's test). ^††^: Significantly different from the vehicle group at *P* < 0.01 (Dunnett's test).

The RXR agonist NEt-3IP showed anti-inflammatory effect in a dose-dependent manner (Figure 3(a)). Oral administration of NEt-3IP at doses of 3 and 10 mg/kg significantly inhibited carrageenan-induced paw edema compared with that of the control group. The control group received 0.5% carboxymethylcellulose solution. NEt-3IP (3 mg/kg) significantly suppressed paw edema at 2 and 3 h after carrageenan injection. NEt-3IP (10 mg/kg) significantly suppressed paw edema at 1, 2, and 3 h after carrageenan injection.  Figure 3(b) shows the effect of the RXR antagonist NS-4TF on the anti-inflammatory effect of NEt-3IP. Intraplantar injection of NS-4TF at a dose of 30 *μ*g/paw significantly reduced the anti-inflammatory effect of NEt-3IP.

### 3.4. Influence of PPAR Antagonists on the Anti-Inflammatory Effect Induced by RXR Agonist

**Figure 4:**
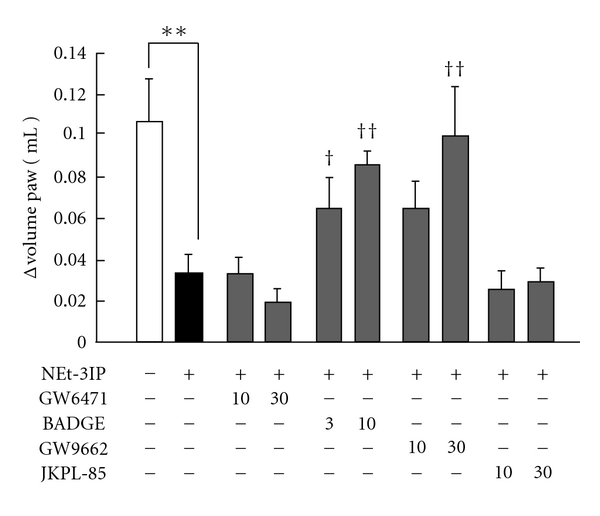
*Influence of PPAR antagonists on RXR agonist and PPAR*γ* agonist-induced anti-inflammatory effects*. NEt-3IP was orally administrated 3 h before carrageenan injection at a dose of 10 mg/kg. Nonselective PPAR antagonist, BADGE was injected intraperitoneal 30 min before carrageenan injection at doses of 10 and 30 mg/kg. PPAR*α* antagonist, GW6471 was injected into subplantar 15 min before carrageenan injection at doses of 10 and 30 *μ*g/paw. PPAR*γ* antagonist, GW9662 was injected into subplantar 15 min before carrageenan injection at doses of 3 and 10 *μ*g/paw. PPAR*δ* antagonist, JKPL-85 was injected into subplantar 15 min before carrageenan injection at doses of 10 and 30 *μ*g/paw. The vehicle group received physiological saline including 10% dimethylsulphoxide. Each column and vertical bar represents the means ± S.E.M. (*n* = 7). **: Significantly different from the control group at *P* < 0.01 (Dunnett's test). ^†^,^††^: Significantly different from the vehicle group at *P* < 0.05 and *P* < 0.01, respectively (Dunnett's test).

Figure 4 shows the influence of GW6471, BADGE, GW9662, and JKPL-85 on the anti-inflammatory effect induced by NEt-3IP. Intraperitoneal injection of the PPAR*γ* antagonist BADGE (30 mg/kg) and intraplantar injection of the PPAR*γ* antagonist GW9662 (3 and 10 *μ*g/paw) significantly reduced the anti-inflammatory effect induced by the RXR agonist. In contrast, intraplantar injection of the PPAR*α* antagonist GW6471 (10 and 30 *μ*g/paw) and the PPAR*δ* antagonist JKPL-85 (10 and 30 *μ*g/paw) did not inhibit the anti-inflammatory effect induced by the RXR agonist. Both antagonists did not affect the paw edema at any time or concentration.

### 3.5. The Combination Effect of RXR Agonist and PPAR*γ* Agonist on Carrageenan-Induced Paw Edema

**Figure 5:**
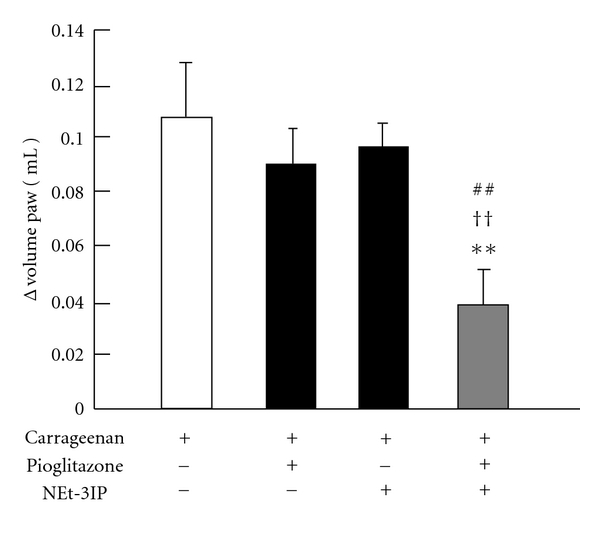
*Effect of combination of RXR agonist and PPAR*γ* agonist on carrageenan-induced paw edema*. Coadministration of pioglitazone (1 mg/kg) and NEt-3IP (1 mg/kg), which showed no inhibition each alone, suppressed carrageenan-induce paw edema compared with control group, pioglitazone (1 mg/kg) and NEt-3IP (1 mg/kg) treated group, respectively. The control group received 0.5% carboxymethylcellulose solution. Each column and vertical bar represents the means ± S.E.M. (*n* = 7). **: Significantly different from the control group at *P* < 0.01 (Dunnett's test). ^††^: Significantly different from the pioglitazone treated group at *P* < 0.01 (Dunnett's test). ^##^: Significantly different from the NEt-3IP treated group at *P* < 0.01 (Dunnett's test).

Figure 5 shows the combination effect of pioglitazone and NEt-3IP on carrageenan-induced paw edema. Coadministration of pioglitazone (1 mg/kg) and NEt-3IP (1 mg/kg) suppressed carrageen-induced paw edema as compared to that of the control group and the groups treated with pioglitazone (1 mg/kg) and NEt-3IP (1 mg/kg).

### 3.6. Influence of Actinomycin D on the Anti-Inflammatory Effect Induced by RXR Agonist and PPAR*γ* Agonist

**Figure 6:**
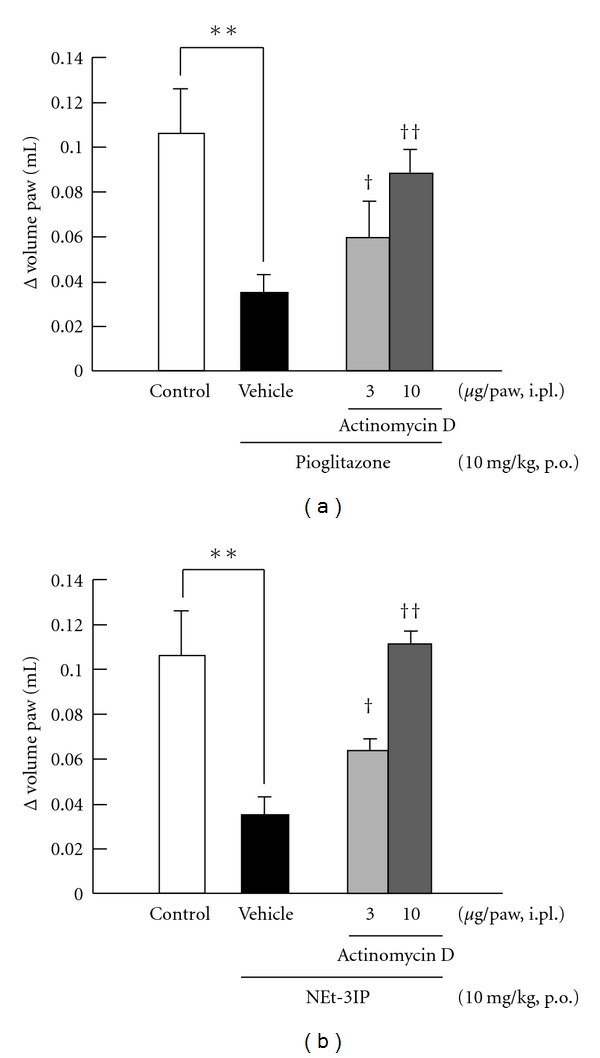
*Influence of RNA polymerase inhibitor on PPAR*γ* agonist and RXR agonist-induced anti-inflammatory effects*. (a) Effect of RNA polymerase inhibitor, actinomycin D on pioglitazone-induced anti-inflammatory effect. Pioglitazone was orally administrated 3 h before carrageenan injection at a dose of 10 mg/kg. Actinomycin D was injected 15 min before carrageenan injection at doses of 3 and 10 *μ*g/paw. The vehicle group received physiological saline including 10% dimethylsulphoxide. (b) Effect of actinomycin D on NEt-3IP-induced anti-inflammatory effect. NEt-3IP was orally administrated 3 h before carrageenan injection at a dose of 10 mg/kg. Actinomycin D was injected 15 min before carrageenan injection at doses of 3 and 10 *μ*g/paw. The vehicle group received physiological saline including 10% dimethylsulphoxide. Each column and vertical bar represents the means ± S.E.M. (*n* = 7). **: Significantly different from the control group at *P* < 0.01 (Dunnett's test). ^†^,^††^: Significantly different from the vehicle group at *P* < 0.05 and *P* < 0.01, respectively, (Dunnett's test).

Figures 6(a) and 6(b) show the influence of the RNA polymerase inhibitor actinomycin D on the anti-inflammatory effect of pioglitazone and NEt-3IP. Intraplantar injection of actinomycin D at doses of 3 and 10 *μ*g/paw significantly reduced the anti-inflammatory effect induced by PPAR*γ* and RXR agonist, respectively, compared with that in vehicle-treated mice.

### 3.7. Influence of Reagents on Edema Induced by Carrageenan

**Figure 7:**
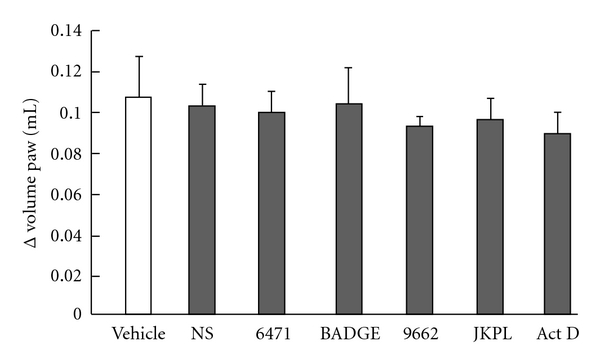
*Influence of reagents on edema induced by carrageenan*. BADGE was injected intraperitoneal 30 min before carrageenan injection at doses of 30 mg/kg. NS-4TF (30 *μ*g/paw), GW6471 (30 *μ*g/paw), BADGE (30 mg/kg), GW9662 (10 *μ*g/paw) and JKPL-85 (30 *μ*g/paw), were injected into subplantar 15 min before carrageenan injection. The vehicle group received physiological saline including 10% dimethylsulphoxide. Each column and vertical bar represents the means ± S.E.M. (*n* = 7).

Figure 7 shows the influence of reagents on edema induced by carrageenan. NS-4TF (30 *μ*g/paw), GW6471 (30 *μ*g/paw), BADGE (30 mg/kg), GW9662 (10 *μ*g/paw), and JKPL-85 (30 *μ*g/paw) have no influence on edema induced by carrageenan.

## 4. Discussion

In the present study, the PPAR*γ* agonist pioglitazone significantly inhibited carrageenan-induced paw edema in mice, and PPAR*γ* antagonist significantly inhibited this suppressive effect. In addition, the effect of PPAR*γ* agonist was prevented by an inhibitor of RNA synthesis. Studies have reported that rosiglitazone or pioglitazone showed anti-inflammatory effect via PPAR*γ* in the carrageenan test [22, 26, 27]; these findings are similar to the findings of our study. A previous study found that the pioglitazone dose showing a significant anti-inflammatory effect was less than that of another study [28]. Therefore, it is suggested that that oral administration of pioglitazone at 3 h before carrageenan injection is a better pathway of administration.

Wan et al. [29] have reported that fasting-induced PPAR*α* activation was strongly inhibited in the absence of hepatocyte RXR*α*. In addition, Wan and Badr [19] have also reported that PPAR*α* agonist did not inhibit carrageenan-induced paw edema in hepatocyte-specific RXR*α*-deficient mice, whereas PPAR*α* agonist reduced carrageenan-induced paw edema in wild-type mice. These results indicate that RXR*α* plays a central role in PPAR*α*-induced effects. Therefore, we hypothesize that the endogenous RXR ligand is involved in PPAR activation. Consequently, we used an RXR antagonist to ignore the influence of the endogenous RXR ligand. As described in the results section, the RXR antagonist significantly reduced the anti-inflammatory effect of the PPAR*γ* agonist. Therefore, we suggest that the endogenous RXR ligand may contribute to PPAR*γ* activation. On the basis of these findings, we speculate that the endogenous RXR ligand and synthetic PPAR*γ* agonist may function synergistically.

Additionally, we investigated the participation of PPAR in the action of RXR. First, we evaluated the effect of the RXR agonist NEt-3IP on carrageenan-induced paw edema. We found that the RXR agonist significantly inhibited carrageenan-induced paw edema in mice and that this effect was significantly inhibited by both RXR antagonist and actinomycin D, an inhibitor of RNA synthesis. RXR binds the nuclear factor kappa B (NF-*κ*B) components p50 and p65 and also inhibits NF-*κ*B transactivation [30]. Moreover, Uchimura et al. [31] have showed that the synthetic RXR agonist Ro47-5944 suppressed LPS-induced inducible nitric oxide synthesis (iNOS) and TNF-*α* mRNA expression. In addition, they also reported that Ro47-5944 suppressed the promoter activity of NF-*κ*B in RAW 264.7 cells. Studies have reported that nuclear translocation of NF-*κ*B was activated by carrageenan injection and that the expression of iNOS and cyclooxigenase-2 (COX-2) observed in paw exudates was induced by carrageenan via NF-*κ*B signalling [21, 23]. Therefore, in the present study, the anti-inflammatory effect of the RXR agonist may be caused via inhibition of NF-*κ*B.

It has been reported that the PPAR/RXR heterodimer can be activated by both RXR and PPAR agonists, either independently or together to cause a synergistic activation [32–34]. Therefore, it is thought that PPAR is involved in the RXR-induced anti-inflammatory effect. However, it is unclear which subtypes of PPAR contribute to the anti-inflammatory effect of RXR agonist. Therefore, we confirmed the effect of subtype-selective PPAR antagonist on RXR agonist-induced anti-inflammatory effect. Our data showed that the PPAR*γ*-selective antagonist, BADGE and GW9662 significantly inhibited the anti-inflammatory effect of the RXR agonist. In contrast, the PPAR*α*-selective antagonist GW6471 and the PPAR*δ*-selective antagonist JKPL-85 did not inhibit the suppressive effect of the RXR agonist. These results confirm that the anti-inflammatory effect of the RXR agonist occurs partially though PPAR*γ* activation. In addition, it is suggested that the endogenous PPAR*γ* ligand may contribute to RXR activation. However, the PPAR/RXR heterodimer can be activated only by RXR agonists via permissive mechanisms [35]. Furthermore, Ijpenberg et al. [36] reported that the RXR/9-cis retinoic acid signalling pathway could selectively bind to peroxisome proliferator response element (PPRE) function and PPAR response element and induce transactivation. Therefore, it is necessary to perform a more detailed in vitro investigation of these functions.

In the case of the PPAR/RXR heterodimer, the binding of the ligand of either receptor can activate the complex, yet simultaneous binding of both ligands is more potent [37, 38]. This raises the question whether the coadministration of PPAR and RXR ligands further enhances the anti-inflammatory effect of either ligand in the carrageenan test. Therefore, we studied whether the PPAR and RXR agonists show synergistic function in the carrageenan test. Administration of either pioglitazone (1 mg/kg) or NEt-3IP (1 mg/kg) showed no effect on carrageenan-induced paw edema; however, the combined administration of these 2 compounds resulted in significant inhibition of carrageenan-induced paw edema. Studies have reported the combined effect of PPAR*γ* and RXR agonists on the chronic inflammatory phase. Desreumaux et al. [39] also reported that simultaneous treatment with the PPAR*γ* agonist rosiglitazone and the RXR agonist LG101305 enhanced the levels of TNF-*α* and interleukin (IL)-1*β* mRNA in the mouse colon. Diab et al. [15] reported that 9-cis retinoic acid showed an effect on experimental autoimmune encephalomyelitis and that this effect was enhanced by the endogenous PPAR agonist 15 deoxy-Δ^12,14^-PGJ_2_. Additionally, Burrage et al. [40] reported that combinatorial treatment with rosiglitazone and the RXR agonist LG100268 inhibits IL-1*β*-induced expression of MMP-1 more effectively than treatment with either individual compound. We showed that RXR and PPAR*γ* agonists could exert a synergistic anti-inflammatory effect during the acute-phase response in vivo.

Wang et al. [41] have reported that LPS, TNF-*α*, and IL-1*β* caused RXR downregulation in mouse kidney cells during the acute-phase response. In addition, Harada et al. [42] have reported that Th1 cytokine induced PPAR**γ** downregulation in human biliary cells. Moreover, it has been reported that RXR and PPAR are suppressed in the liver and heart during the acute-phase response [43]. Furthermore, Wan et al. [44] reported that the expression levels of both PPAR and RXR mRNA decrease in tissues, including the animal model of liver inflammation. These findings indicate that each agonist of PPAR*γ* and RXR may be important for the action of therapeutic drugs on inflammatory diseases.

In conclusion, we found that PPAR*γ* agonist-induced anti-inflammatory effects were reversed by RXR antagonist. Thus, we showed that the endogenous RXR ligand might contribute to the PPAR*γ* agonist-induced anti-inflammatory effect.
